# The Development of Instruments to Detect Indicators of Behavioral Changes in Therapeutic Communities: A Clinical Case Study

**DOI:** 10.3389/fpubh.2019.00319

**Published:** 2019-11-22

**Authors:** Stefania Cristofanelli, Agata Ando', Laura Ferro

**Affiliations:** ^1^Department of Social Sciences and Humanities, University of Valle D'Aosta, Aosta, Italy; ^2^Department of Psychology, University of Turin, Turin, Italy

**Keywords:** psychological assessment, therapeutic communities, psychological functioning, behavioral problems, adolescence

## Abstract

Clinicians involving in the treatment of adolescent patients should use a valid and efficient psychological assessment. The evaluation of the efficiency in clinical interventions may provide helpful information in terms of cost-effectiveness and may contribute to increase the quality and efficacy of the public services. Despite the importance of clinical and therapeutic interventions, we may observe several aspects limiting the chance in using them. For example, the neuropsychiatry context due to heterogeneous users (such as children and adolescents) makes the replicability of clinical trials difficult in terms of results. Thus, efficient clinical programs and interventions—potentially able to identify specific and long-term effects—need to be defined. In clinical contexts (i.e., therapeutic communities). It should be a priority both to manage aspects of emergency/urgency we may observe in adolescents, and to focus on those aspects placed on a timing dimension. The current study reports a description of innovative measures developed specifically for assessing adolescent patients and for tracking psychological features and behavioral changes. Furthermore, a clinical case is examined by using a multimethod assessment including such innovative measures. Clinical implications are discussed. The development and sharing of “assessment cultures” among professionals should represent a priority in improving the effectiveness of therapeutic communities.

## Introduction

The therapeutic community (TC) is an intensive and comprehensive treatment model, developed for treating adults and adolescents with psychopathology. The core goal of TCs is to promote a healthy lifestyle, and to identify those areas for changes such as negative behaviors leading to no adaptive conditions (1, 2). In TCs, individuals are distinguished along dimensions of psychological dysfunction and social deficits; indeed, a considerable number of patients never has acquired conventional lifestyles, and specific educational deficits are marked, and several values are either missing or unpursued. Most often, patients derive from a socially disadvantaged sector where dysfunctional behaviors are considered as a social response rather than a psychological disturbance (3).

The Community of Communities (C. of C.) is a quality improvement and accreditation programme for Therapeutic Communities in the UK and overseas. The Community of Communities' activities support members to meet the highest standards of TC practice through a process of self- and peer-review. Members of TCs work with adults and children with a range of multiple needs including personality disorders, offending behaviors, learning disabilities, addictions, and severe mental illness. The C. of C. combines the application of Service Standards for a quality improvement with the benefits of a peer-support network. The Service Standards for Therapeutic Communities for Children and Young People are decided every year and are applied through a process of self- review, and external peer review where members visit each other's services (4).

The evaluation of indicators of the change of outcomes and processes in clinical interventions is becoming increasingly important in Italy, especially in the public health context. Therapeutic Communities for children and adolescents may be described as a set of heterogeneous services, based on different organizational procedures, driven by a set of values and practice standards, accruing from multiple theoretical foundations and numerous service delivery traditions. Therefore, the services are significantly different from one another and cannot be described by applying an unitary model with a single set of definite outcomes. At this point, the necessity that the TC staff works toward greater explicitness, consistency, and cohesiveness may be urgent and compelling (5, 6). The main goal of the current study was to identify and develop specific tools in order to assess behavioral changes in adolescent therapeutic communities. Specifically, we conducted a single case study using a multimethod assessment including measures for evaluating specific psychological dimensions associated to behavioral changes over time.

### Measures Development

Psychotherapists, psychiatrists, and psychologists with a long clinical experience in therapeutic communities, and expert researchers in the psychological assessment field^1^ discussed and worked on the development of specific measures for assessing adolescents in therapeutic communities; specifically, after revising the scientific literature on residential care contexts and adolescent therapeutic communities [e.g., (7, 8)], and previous clinical data, they identified those measures (to include in the assessment) that were potentially able to evaluate specific psychological dimensions [i.e., belongingness and motivation, self-esteem and self-regulation, and interpersonal relationships underlying possible related observable behaviors; (9–11)]. The description of the measures is reported below.

#### The Medical History Form

The neuropsychiatrist completes the medical history form, a tool including patient's anamnesis and information on the family system.

#### The Daily Behaviors Logbook

By using the behaviors logbook, health educators report daily specific behavioral patterns in a patient, as follows. (A1) *Structured group activities* (i.e., eating occasions, excursions, and creative laboratories); the response options for each question are two: *attending, not attending*; (A2) *How behaviors occur in those structured activities*. The response options are the following: *negatively, positively*. (B) *Psychological crisis types*. The responses options are the following*: aggressive, dysphoric*. (C) *Family relationships* (i.e., meeting and calling his/her relatives); the response options are the following: n*egatively, positively*. (D) *Other behaviors and events* (i.e., hospitalization, police intervention, school attendance; work/traineeship attendance, sickness, request for the drug therapy; escape from the community, and personal hygiene); the response options are three: *yes, no, I do not know*. (E) *Daily Mood*; the response options are the following: *worried, serene, cheerful, depressed, hyperactive, angry, sad, passive, upset, nervous, anxious*.

#### The Therapeutic Project Report

Clinicians complete the therapeutic project report describing the patient's behavior and overall psychological functioning in following contexts. (A) *Homecoming*s (i.e., homecoming events; desire to return to home, negative homecomings, returns to the community soon after the homecoming are positive). (B) *School management* (i.e., motivation to go school, low academic achievement, school attendance, good peer relations). (C) *Living in the therapeutic community* (i.e., good interactions with educators, conflictual interpersonal interactions, proactive behaviors). (D) *Family relationships* (i.e., positive meeting/call to his/her relatives, presence of family members in the patient's life, negative interactions with family members, family members cause severe discomforts in the patients). (E) *Traineeship/work context* (i.e., work motivation, good academic achievement, poor work attendance, negative relations with his/her coworkers). (F) S*ociability and interpersonal relations* (i.e., frequency of friendly and positive relationships outside the community, desire to have friendly and positive relationships outside the community). (G) *Romantic Relationships* (i.e., desire to have a romantic relationship; being in a romantic relationship). (H) *Personal Care* (i.e., good personal hygiene, autonomy in his/her personal care, adequate clothing, clean clothing. (I) *Management of his/her physical and mental health* (i.e., attendance at clinical interviews; adherence to the medication regimen; request for the drug therapy. (L) *Money management* (i.e., efficient and adequate money management). The response options for all aforementioned items are the following: *never, seldom, sometimes, often, always*. (M) *Ability to control impulsive behaviors* (i.e., level of emotional and cognitive resources, negative emotions, experiences of environmental stress). (N) *Affectivity* (i.e., awareness and understanding of emotions, behavior when experiencing emotions, regulation of emotions effectively, tendency to have negative secondary emotional responses to one's negative emotions). (O) *Self-perception and Interpersonal relationships* (i.e., level of introspective ability, narcissistic features, negative self-perceptions, self-esteem, mental representations of other people, inflexibility in relationships, autonomy in relationships). (P) *Reality testing* (i.e., efficiency of reality testing). The response options for each item included in *Ability to control impulsive behaviors, Affectivity, Self-Perception and Interpersonal relationship*, and *Reality testing* domains are the following: *Very low, Low, Moderate, High, Very high*. The therapeutic project report is completed quarterly.

#### Clinical Supervision Report

The Clinical Supervision Report includes the exchange of qualitative information between clinicians, on patient's behaviors and interactions (e.g., with family, with clinicians and health educators, with coworkers, with peers). Clinicians, quarterly, complete this report. The Clinical Supervision Report aims to include an exchange of information between clinicians on specific areas of the psychological functioning in patients. It represents a clinical discussion on the psychological functioning of the patient according to multiple points of view of all clinicians working with adolescents. Information reported by The Clinical Supervision Report has practical and clinical significance given that it may be shared with the health educators taking charge of users daily.

Information obtained by the aforementioned tools is collected and organized with Google forms.

## The clinical case study: background and results

### The Medical History Sheet

Pietro^2^ is a 16-year-old male. His father is currently unemployed due to instable health conditions (he is affected by cardiopathic, diabetic, asthmatic problems); he is described by Pietro as a detached and not authoritative figure, but previously aggressive toward his family. Some members of the original family of Pietro's father are characterized by aggressive and defiant behaviors. Pietro's mother is a worker. She usually shows feelings of infantile reactions to frustrating conditions. She is not able to assume the “parent role” having established an equal relationship with Pietro (Pietro seems to be considered as partner or a little brother by his mother). Pietro's mother and maternal grandfather are affected by severe affective disorders. Pietro's older brother (he is 22 years old), lives with his parents and he is considered by the family as “the only right and nice son.” Currently, he shows severe withdrawal behaviors.

Pietro's family seems to have failed in providing for Pietro emotional and physical needs; Parents are characterized by clearly immature and incompetent personalities. Family communication patterns severely increased the dysfunctional expression of feelings in Pietro. Pietro growing up in such family developed low self-esteem and felt that his needs were not important or were not taken seriously by his family. When was 5 years received a diagnosis of intellectual disability showing deficits in general mental abilities such judgment and learning from experience. The deficits resulted in impairments of the overall adaptive functioning. During his early adolescence, Pietro showed serious behavioral and emotional disorders displaying a pattern of disruptive and violent behavior, and problems in following rules; inaddition, Pietro used cannabis showing a severe distress. Therefore, he attended several residential child-care institutions. Subsequently, Pietro received a diagnosis of Bipolar I disorder by neuropsychiatrists, in the current adolescent therapeutic community^3^.

### The Daily Behaviors Logbook

We grouped daily information and data in three times (see Table 1): T1 (from March 2017 to June 2017), T2 (from July 2017 to November 2017), T3 (from December 2017 to February 2018). In Table 1, we reported the percentage of the response options for each question, during the three times.

**Table 1:** The daily behaviors logbook.

	**Scores**		
	**Frequency %**		
**The daily behaviors logbook**	**T1 (%)**	**T2 (%)**	**T3 (%)**
**(A1) Structured group activities (attending)**			
Eating occasions	96	97.2	84.3
Excursions	38	41	31
Creative laboratories	30	31.8	21.1
**(A2) How behaviors occur in those structured activities (positively)**			
Eating occasions	89.5	95.5	68.4
Excursions	40.4	34.1	31.6
Creative Laboratories	17.5	22	15.8
**B) Psychological crisis type (occurrence)**			
Aggressive	0	2.4	0
Dysphoric	0	0	1
**(C) Family relationships (positively)**			
Meeting and Calling his/her relatives	29.8	31.7	15.8
**(D) Other behaviors and events (yes)**			
Hospitalization	0	0	0
Police Interventions	0	0	0
School Attendance	1.8	17.1	31.6
Sickness	3.5	2.4	0
Request for the drug therapy;	56.1	9.8	5.3
Escapes from the community	4	0	0
Personal Hygiene	64	87.8	78.9
**(E) Daily Mood (presence of mood)**			
Worried	5.3	4.9	0
Serene	61.4	78	84.2
Cheerful	38.6	56.1	52.6
Depressed	1.8	0	0
Hyperactive	7	17.1	5.3
Angry	10.5	9.8	5.3
Sad	5.3	2.4	10.5
Passive	1.8	0	0
Upset	19.3	19.5	10.5
Nervous	15.8	12.2	21.1
Anxious	10.5	9.8	0

*Data are grouped in three times. The frequencies percentage of response options for each question is included*.

The presence of Pietro during eating occasions, excursion and creative laboratories is more frequent at T2 than T1 and T3; Pietro's behaviors seem to be more adequate and positive regarding the eating occasions and creative laboratories during T2 than T1 and T3. Differently, a less positive attitude occurs during the excursions at T2 than the other two times. Pietro reports aggressive crises only at T2. He calls more frequently his relatives at T2 than T1 and T3. Pietro's school attendance is higher at T2 respect to the other two times, while some episodes of sickness result to be more frequent at T1. It is noteworthy that the frequency of requesting for psychiatric drugs decreases in T3. Pietro escapes from the community exclusively during T1. Personal hygiene occurs especially at T2. The mood that Pietro shows more frequently in all three times is the “serene mood,” while the moods less reported by Pietro are “worried,” “passive,” “depressed,” and “anxious.”

### The Therapeutic Project Report

In Table 2, we reported the response options for each question, during the three times. Pietro returns more frequently to home at T3 (also his desire to go to home is especially reported during T3). Sometimes at T3, we can observe the occurrence of negative homecomings and positive returns to the community soon after his homecomings. Motivation to go to school, low academic achievement and school attendance are often present at T3, while good relations with peers occur sometimes (at T3). Pietro shows frequently good interactions with educators, and proactive behaviors in the TC context at T3, while conflictual interpersonal interactions occur sometimes in all three times. Furthermore, we can observe sometimes at T3, the constant presence of family members, negative interactions between Pietro and his family, and discomforts in Pietro caused by family members' actions. Positive meetings/calls between Pietro and his family are reported as very frequent at T3. Frequency of friendly and positive relationships outside the community are seldom at T1 and T2 and the desire to have friendly and positive relationships outside the community occur frequently in all three times. Instead, the desire to have a romantic relationship is less evident at T2. We can observe the overall efficient personal care in all three times despite the autonomy in his personal care is seldom at T2 and T3. Pietro always attends clinical interviews and follows medication regimen at T3, and requests drug therapy sometimes at T3. The efficiency in money management occurs only sometimes at T3. Emotional and cognitive resources, negative emotions, and some experiences of environmental stress are moderately present at T3. The awareness and understanding of emotions are low at T3. Some behaviors when Pietro experiencing emotions, regulation of emotions effectively and the tendency to have negative secondary emotional responses to one's negative emotions are moderate at T3. Narcissistic features are present at T3 and seem to be in contrast to the self-esteem that is reported as very low at the same time (or T3). Pietro reports a lower level of introspectively ability at T1 than T2 and T3. A very low negative self-perception is reported at T3. Mental representations of other people are moderate in all three times. We can observe a high inflexibility in relationships at T1 and T2, while it is very low at T3. The autonomy in relationships is low and very low in the three times, while the efficiency of reality testing is high at T3.

**Table 2:** The Therapeutic project report.

**The Therapeutic project report**	**T1**	**T2**	**T3**
**(A) Homecomings**			
Homecoming events	N/A	Sometimes	Always
Desire to return to home	N/A	Sometimes	Often
Negative homecomings	N/A	Seldom	Sometimes
Returns to the community soon after the homecoming are positive	N/A	Seldom	Sometimes
**(B) School management**			
Motivation to go to school	Missing	Missing	Often
Low academic achievement	Missing	Missing	Often
School attendance	Missing	Missing	Often
Good peer relations	Missing	Missing	Sometimes
**(C) Living in the therapeutic community**			
Good interactions with educators	Often	Sometimes	Often
Conflictual interpersonal interactions	Sometimes	Sometimes	Sometimes
Proactive behaviors	Seldom	Seldom	Often
**(D) Family relationships**			
Positive meeting/call to his/her relatives	Often	Always	Often
Presence of family members in the patient's life	Seldom	Sometimes	Sometimes
Negative interactions with the family members	Sometimes	Seldom	Sometimes
Family members causes severe discomforts in the patients	Seldom	Sometimes	Sometimes
**(E) Traineeship/work context**			
Work motivation	N/A	N/A	N/A
Good academic achievement	N/A	N/A	N/A
Negative relations with his/her coworker	N/A	N/A	N/A
**(F) Sociability and interpersonal relations**			
Frequency of friendly and positive relationships outside the community	Seldom	Seldom	Sometimes
Desire to have friendly and positive relationships outside the community	Often	Often	Often
**(G) Romantic Relationships**			
Desire to have a romantic relationship	Often	Seldom	Often
Being in romantic relationship	Never	Never	Missing
**(H) Personal Care**			
Good personal hygiene	Sometimes	Sometimes	Often
Autonomy in his/her personal care	Sometimes	Seldom	Seldom
Adequate clothing	Often	Sometimes	Often
Clean clothing	Sometimes	Sometimes	Often
**(I) Management of his/her physical and mental health**			
Attendance at clinical interviews	Often	Often	Always
Adherence to the medication regimen	Often	Often	Always
Request for the drug therapy	Seldom	Sometimes	Seldom
**(L) Money management**			
Efficient and adequate money management	Missing	Often	Sometimes
**(M) Ability to control impulsive behaviors**			
Level of emotional and cognitive resources	Moderate	Low	Moderate
Negative emotions	Moderate	High	Moderate
Experiences of environmental stress	Low	Low	Moderate
**(N) Affectivity**			
Awareness and understanding of emotions	Moderate	Low	Low
Behavior when experiencing emotions	Moderate	High	Moderate
Regulation of emotions effectively	Moderate	High	Moderate
Tendency to have negative secondary emotional responses to one's negative emotions	Low	Moderate	Moderate
**(O) Self—perception and Interpersonal relationships**			
Level of introspective ability	Very low	Low	Low
Narcissistic features	Moderate	High	High
Negative self- perceptions	Moderate	High	Moderate
Self-esteem	High	Very low	Very low
Mental representations of other people	Moderate	Moderate	Moderate
Inflexibility in relationships	High	High	Very low
Autonomy in relationships	Very low	Very low	Low
**(P) Reality testing**			
Efficiency of reality testing	Moderate	Moderate	High

### The Clinical Supervision Report

All qualitative information on patient's behaviors and interactions, between clinicians is included in Table 3. Table reported information concerning Pietro's interactions with his family, with clinicians and health educators, and peers.

**Table 3:** The clinical supervision report.

**T1 (from March 2017 to June 2017)**
Pietro's father, currently unemployed, made a serious car accident during which a man died. The car accident caused a serious discomfort in Pietro and represented for him a traumatic event that has worsened relations with his father. Pietro's father is depressed but in the past, he was very aggressive toward his family members. Pietro has an older brother. Both Pietro's father and brother seem to be characterized by detached relationships, emotional unavailability, and psychological immaturity; they are emotionally void and burned without coping strategies able to manage their emotional and psychological needs maintaining negative patterns of behavior due to lack of self-awareness. Pietro's mother seems to use her child to get her emotional needs. The relationship between Pietro and her mother appear to be dependent and entangled; Pietro slept in the same bed with her mother until adolescence. Toward the mother, Pietro shows both aggressive and dependent behavior. When Pietro was 5 years received a diagnosis of intellectual disability characterized by significant limitations in both intellectual functioning and adaptive behavior. He was supported by therapeutic program and subsequently was included in two TCs where he referred to have experienced sexual abuses (sexual abuses were not confirmed by medical examinations). Later, Pietro received the diagnosis of a bipolar disorder because of extreme mood swings including emotional “highs” and “lows.” In the community, Pietro shows a proactive behavior and engages in daily activities. He has a good personal care and participates in those activities organized by the community such as sports activities and trips. When interactions are not mediated by “external structured frames” (such as the TC), he usually shows antisocial behaviors and specifically acts characterized by covert and overt hostility and intentional aggression toward others. Overall, significant limitations in both intellectual functioning and in adaptive behaviors contribute to increase difficulties in understanding the demands external to the TC.
**T2 (from July 2017 to November 2017)**
Initially Pietro tried several times to escape from the community. Especially during this second period, (T2) escapes decreased. Clinicians and health educators believe that relations with the community are becoming stable and productive. During the activities outside the TC, Pietro shows collaborative behaviors. However, when he is in the community with others shows more frequently antisocial and non-adaptive behaviors. In the TC Pietro is described as attention—seeker, showing aggressive crises in order to elicit attention; this type of behavior seems to be associated to primitive and symbiotic relationship with her mother. Clinicians believe that it is essential to accept such “emotive requests” by Pietro in order to move from a condition of *infantile omnipotence* to a *non-omnipotence condition*. The sense of omnipotence arises from the fundamental misapprehension of reality, which is central to the period of primary narcissism, during which the infant hallucinates its original love-object and overestimate the power of wishful thinking. After meeting his family (about once a month), Pietro is described as apparently calm and serene.
**T3 (from December 2017 to February 2018)**
Pietro's interpersonal relationships continue to be immature. He shows attention-seeking behaviors and does not try to manage his feelings of frustrations especially when others fool with him. He seems to be always looking for male adult figures in which to “mirror” him-self. The “*male* argument” represents in Peter's life an aspect linked to feelings of fear and anguish. Specifically, the car accident caused by his father contributed to the social isolation of his family. The lacking of a male figure is evident in “looking for male figures” (such as male health educators toward which it seems to show dependence and intrusiveness). Pietro engages in dependent and submissive behaviors that are designed to elicit care-giving behaviors in others. Overall, his dependent and infantile behavior may be considered as being “clingy” or “clinging on” to others. Pietro consistently seems to express to feel abandoned when health educators are not involved in his daily activities- Pietro struggles every day with his emotions and episodic bouts of self-loathing. Pietro shows more difficulties with social adjustment, and report problems with friendships/peers, and manifest behavior problems (especially outside the community). For example, Pietro is a swaggering, in an attempt to satisfy needs of others when attending groups with “swaggering” people. He changes depending on people that are around. However, he seems to accept more than before rules and limitations given by the TC; indeed, he follows rules, structured activities and shows a good daily personal care.

## Discussion

The current study aimed to provide a valid contribute for developing specific instruments able to assess the psychological functioning and behavioral changes in adolescent patients of therapeutic communities. Few studies have been conducted to establish how therapeutic communities work to produce positive outcomes, not reporting the description of those tools included in the psychological assessment (12). Behavioral changes are crucial to improve those voluntary behaviors over which the person has at least a degree of control (10, 12). Recovery or improvement thus requires the individual takes active steps to change unhelpful habits or entrenched patterns of behavior. Therefore, the necessity that the TC staff works by using specific and *ad hoc* measures should be considered as a priority. We conducted a single case study using an innovative multimethod assessment including new measures for evaluating psychological features related specific behavioral changes. This multimethod assessment approach may provide useful information on specific psychological dimensions (13–15); also, data and information obtained by these new measures may help clinicians to establish individual treatment approaches.

In structured group activities, Pietro reports a constant attendance but the quality and frequency of his behavior decreases at T3. There is an overall deterioration from T2 to T3. However, staying in the community may lead to daily obstacles that Pietro does not cope: in fact, sometimes he shows aggressive crises at T2, that may represent difficulties in maintaining relationships with others (especially, after some months from the community admission). Relations with his family members are stable, although we can observe a “worsening” in the psychological functioning and well-being at T3, probably subsequently to one or more meetings with his family system. Distress experiences occur intensely only during the meeting between Pietro and his family; differently when Pietro calls his parents arises a situation in which the phone assumes the function like a “wall” protecting Pietro from the impact of family's actions. Also, we can observe a stable attendance in school activities at T3: we can speculate that after the homecomings despite some difficulties, Pietro adheres to the structured context of the community and follows its rules (such as to go to the school constantly). It is noteworthy that the request for pharmacological therapy drastically decreases at T3, probably for the improvement of the awareness related to needs and for the improvement of the capacity in managing several problems. There are also some aspects associated with restless and nervousness. At T3 Pietro shows the desire to return to home but at the same times, we can observe that negative returns occur. Overall, we can note that when interactions are not mediated by “external structured frames” (such as those of the TC), Pietro usually shows no adaptive behaviors. For example—at home with his family—he is absorbed by a “world without rules,” lacking in psychological containment and, therefore, emotion dysregulation and specifically, difficulties in controlling impulses increase sometimes leading to substance abuse and no conventional behaviors. We may infer that there is a difficulty in modulating emotions without an external containment that causes some dissociative components. Pietro reports a disruption and discontinuity in the normal integration of consciousness, identify, emotions, and perceptions. We can speculate on the presence of dissociative features can potentially may disrupt every area of psychological functioning. Pietro seems to experience unbidden intrusions into awareness and behavior, with accompanying losses of continuity in subjective experiences and inability to access information or to control his mental functions that normally are readily amenable to access or control. These aspects, in fact, emerged from that behavior defined as “chameleonic” resulting in a lacking of internalization of rules and mental states of others. His behavior changes drastically according to the different contexts; he alternates his being a “demon” or a “good boy,” according to the people around him. Therefore, we can observe a discontinuity in his behaviors every time he returns to the community, after his homecomings. Pietro's behavior is not linear: when the context changes, Pietro modifies drastically his behavior assuming different roles with different temperaments.

## Concluding Remarks

The current study presents some limitations. Some data on Pietro's behavior are missing.

Furthermore, the study provides only qualitative data in order to address clinicians to use specific information able to establish a correct and efficient treatment. Although the most prominent critique of single case study is the issue of external validity or generalizability, our findings may improve the knowledge in understanding the psychological functioning. Therefore, we chose to report a single case study in order to examine thoroughly that qualitative information related to psychological functioning that could not be obtained through a quantitative—multiple cases study (16). In conclusion, in future it could be useful to add to the aforementioned tools, a *Daily Emotions Logbook* completed by the patient, in order to obtain important information based on the point of view of the patient.

## Ethics Statement

All procedures performed in studies involving human participants were in accordance with the ethical standards of the institutional and/or national research committee and with the 1964 Helsinki declaration and its later amendments or comparable ethical standards. The protocol was approved by Tiarè—Servizi per la Salute Mentale, Via Berthollet, 44, 10125 Torino, Italy. Project Title: Lo sviluppo di strumenti atti a valutare i cambiamenti del comportamento, nelle comunità terapeutiche. Written informed consent was obtained from the parents of the participant for the study and for the publication of this case report (i.e., The development of instruments to detect indicators of behavioral changes in therapeutic communities: A clinical case study).

## Author Contributions

SC conceived and supervised the study and reviewed the manuscript. AA coordinated the study, reviewed the data, performed the data analysis, and wrote the manuscript. LF helped interpret the data and reviewed the manuscript.

### Conflict of Interest

The authors declare that the research was conducted in the absence of any commercial or financial relationships that could be construed as a potential conflict of interest.

## Footnotes

^1^The current study was conducted in Piedmont (Italy).

^2^Pietro is a fictitious name.

^3^This therapeutic community for adolescences is located in a small city of Piedmont.
